# Recent trends and variations in general practitioners’ involvement in accident care in Switzerland: an analysis of claims data

**DOI:** 10.1186/s12875-020-01170-5

**Published:** 2020-06-05

**Authors:** Marc Höglinger, Fabio Knöfler, Rita Schaumann-von Stosch, Stefan M. Scholz-Odermatt, Klaus Eichler

**Affiliations:** 1 Winterthur Institute of Health Economics, Zurich University of Applied Sciences, Gertrudstrasse 15, 8401 Winterthur, Switzerland; 2 XUND – Bildungszentrum Gesundheit Zentralschweiz, Alpnach, Switzerland; 3 SUVA – Swiss National Accident Insurance Fund, Lucerne, Switzerland; 4 SGTV – Swiss Association for Traumatology and Insurance Medicine, Bern, Switzerland

**Keywords:** Accident care, Trauma care, General practitioner, Emergency department, Patient behavior, Primary care, Health Services research, Claims data

## Abstract

**Background:**

As in other countries, there is concern and some fragmentary evidence that GPs’ central role in the Swiss healthcare system as the primary provider of care might be changing or even be in decline. Our study gives a systematic account of GPs’ involvement in accident care from 2008 to 2016 and identifies changes in GPs’ involvement in this typical field of primary care: how frequently GPs were involved along the care pathway, to what extent they figured as initial care provider, and what their role in the care pathway was.

**Methods:**

Using a claims dataset from the largest Swiss accident insurer with two million accident cases, we constructed individual care pathways, i.e., when and from which providers patients received care. We calculated probabilities for the involvement of various care provider groups, for initial care provision, and for the role of GPs in patients’ care pathways, adjusted for injury and patient characteristics using multinomial regression.

**Results:**

In 2014, GPs were involved in 70% of all accident cases requiring outpatient care but no inpatient stay, and provided initial care in 56%. While involvement stayed at about the same level for accidents occurring from 2008 to 2014, the share of accidents where GPs provided initial care decreased by 4 percentage points. The share of cases where GPs acted as sole care provider decreased by 7 percentage points down to 44%. At the same time, accident cases involving care from an ED at any point in time increased from 38 to 46% and the share receiving initial care from an ED from 30 to 35 percentage points – apparently substituting for the declining involvement of GPs in initial care. GPs’ involvement in accident care is higher in rural compared to urban regions, among elderly compared to younger patients, and among Swiss compared to non-Swiss citizens.

**Conclusions:**

GPs play a key role in accident care with considerable variation depending on region and patient profile. From 2008 to 2014, there is a remarkable decline in GPs’ provision of initial care after an accident. This is a strong indication that the GPs’ role in the Swiss healthcare system is changing.

## Background

As in many other European countries, general practitioners (GPs) have an essential function in the Swiss health care system as the main provider of ambulatory care [1, 2]. General practitioners include physicians in private practice certified as general practitioners, specialists in general internal medicine, and physicians without a specialty qualification. With 83 GPs per 100,000 inhabitants, Switzerland has a high GP density when compared with other countries [3]. Besides seeing patients by appointment and during daytime, they usually provide out-of-hours emergency care, often by participating in local emergency networks operated by GPs themselves.

Patients traditionally first turn to their personal GP when in need of care, even in the case of an emergency. If necessary, GPs then refer patients to a hospital emergency department (ED) or to some other specialized (secondary) care provider. However, there is no general formal gatekeeping system that regulates access to hospitals or specialized care for patients in Switzerland. Still, GPs usually function as gatekeepers in practice and health insurance schemes employing GPs as the central point of contact are increasingly popular as they come with reduced premiums.

Accident insurance in Switzerland is organized separately from health insurance, which only covers non-accident related health care. Both accident insurance nd health insurance are compulsory. However, while individuals choose and pay their health insurance provider on their own, accident insurance is paid for by employers and employees jointly. It covers both occupational and non-occupational accidents. Non-working persons or marginally employed persons need to obtain non-occupational accident coverage from their health insurance provider.

All health care services provided by medical doctors (GPs and specialists) as well as hospital outpatient services covered by the mandatory health insurance are reimbursed on the basis of a nationally uniform tariff system. For hospital inpatient care and rehabilitation care, similar tarif systems are in place. Since 2012, hospital inpatient care has been paid on a case-based lump sum basis (Swiss-DRG), leading to some significant changes such as a decrease in average hospital stay [3]. Reimbursement for accident care follows in general the same regulating principles and tariffs. However, accident care is fully reimbursed to patients without any deductibles and there are no restrictions as to where patients seek care (as compared to health insurance where gatekeeping schemes are available).

While GPs’ importance is particularly acknowledged in the long-term provision and coordination of care for chronically ill and multimorbid patients, GPs also treat a substantial share of patients who have suffered an accident. Approximately 15% of all GP consultations are related to accidents [4]. However, there is no data on the actual share of accident care that GPs provide relative to other providers, in particular hospital EDs. In addition, not much is known about the precise role GPs play, i.e. how often they act as initial care provider, are the sole care provider, refer patients to other providers, or act as a follow-up care provider only. Accident patients are a very heterogeneous group, as their injuries can vary greatly in nature, cause, location and severity. Approximately one-third of all accidents are related to patients’ occupational activities and two-thirds are leisure accidents [5]. Both occupational and non-occupational accidents mostly lead to minor to moderate injuries such as superficial wounds and bruises or sprains and strains, injuries which could be treated by a GP and which, in general, do not require care in an outpatient ED.

The role of GPs in Switzerland has been changing in recent years in various regards. First, patients increasingly have been seeking care directly in EDs, also for minor issues for which a GP could provide suitable care [6–8]. Potential explanations for this development are patients’ ignorance of emergency services operated by GPs, the absence of a personal GP, the geographical proximity to a hospital, or the subjectively-felt urgency of treatment [9].

Second, there is a relatively low and continuously decreasing number of GPs that are based in rural areas [10], a problem that will be accentuated in the near future due to a lack of young GPs that could replace an aging GP population [11]. Third, treatments that GPs do and do not provide seem to have changed. According to a survey of GPs, they provided less trauma-related care in 2012 compared to 1993 and the share of GPs that never perform surgical interventions increased from zero to 9% – however, with large regional variations. Compared to their urban counterparts, GPs in rural areas were more likely to perform a broader spectrum of treatments [12]. Also, GPs are no longer required to cover traumatology during continuous medical education. In sum, these developments may have led to a shift in the role of GPs in general care and particularly in accident care. However, the precise characteristics and the extent of this shift are not well understood. This is a gap that our study addresses.

The aim of our study is to identify the degree of involvement and the actual role of GPs in accident care, the corresponding changes in recent years, and regional variations and differences depending on patients’ characteristics. Our analysis is an important first step in gaining a clear understanding of the scope of the changes and provides guidance in exploring potential driving factors. Also, it might help to partly explain increases in average treatment costs. Various studies have shown that GPs provide emergency services at lower costs than EDs, partly due to the less intensive use of diagnostic measures [13, 14]. Patient satisfaction with GPs’ emergency care is, nonetheless, at a very high level and no different to EDs [15]. Additionally, the study might help to identify topics in traumatology that should be taught to young GPs.

## Methods

### Data

We analyzed claims data from 2008 to 2016 for accidents registered from 2008 to 2014 with the Swiss National Accident Insurance Fund (SUVA), Switzerland’s largest accident insurance provider covering about half of the active working population. From all accidents over this seven-year period, we excluded cases of victims living abroad or receiving any medical treatment abroad, because this study is about accident care in Switzerland. Also, accident victims under the age of 18 and above retirement age (65) were excluded, in order to improve the homogeneity of the study subjects. For the same reason, we excluded accidents with dental lesions as main injury, which have quite different care pathways than other accidents. Furthermore, cases were excluded for which the accident happened more than 3 months prior to registration, since these cases are not typical for primary care. Cases without any treatment costs were excluded as well as cases for which none of the medical provider groups under consideration was involved (i.e. only pharmaceutical services). We excluded accidents with an inpatient stay at any point during the care pathway from our main analyses because of only partially reported data on patients’ beginning of inpatient stays prior to 2014. However, we do report analysis results including inpatient cases, where possible, in the Supporting Information S 5.

Our data contains the year of the accident, information about the point in time of the accident (day-of-week and time-of-day), the injury type and anatomical location as reported by the insured person or their employers in the accident report form, the patient’s gender, citizenship (Swiss or foreign), age, and place of residence using an urban-intermediate-rural typology according to FOS classification [16]. We have information on the reimbursed daily allowances, which permits us to indirectly infer the injury severity. An incapacity duration exceeding three working days entitles the employer to a compensation through daily allowances and hence is reflected in the data.

From the billing data of all healthcare services provided that are related to one particular accident, we extracted the direct medical costs incurred in the 2 years following the accident, as well as the date of the first patient contact of specific health care provider groups such as general practitioners, medical specialists, or hospitals. Hospital care is differentiated according to outpatient and inpatient service. Inpatient service is defined as care that involved an overnight stay of the patient.

Provider groups are defined as follows: GPs include physicians in private practice certified as general practitioners, specialists in general internal medicine, and physicians without a specialty qualification. The GP category also includes, to a very small degree, emergency walk-in centers, i.e. clinics with extended availability but no beds, which are operated mostly by GPs (locally called “Permanence”). Medical specialists include orthopedic surgeons, ophthalmologists, non-orthopedic surgeons, and other specialists. We did not consider radiologists, pharmacists, physiotherapists, dentists or rescue services in the construction of care pathways because they generally have a supportive function beside one or more main care provider. ED outpatient includes hospital emergency departments providing outpatient care, i.e. care without an overnight stay of the patient.

We have information about the date of the first patient contact of a particular provider group, but not about the number and timing of subsequent contacts with the same provider or other providers of the same provider group. However, the date of the first patient contact allows us to identify who figured as initial care provider and to reconstruct individual care pathways: the first provider group providing care, the second provider group, the third provider group, etc. With this information, we can distinguish care pathways, for instance, cases where GPs acted as sole care provider from others where GPs provided initial care and then referred a patient to the hospital.

If more than one group of providers had their first patient contact on the same day, we assumed the following plausible ordering of the care sequence for trauma patients to unequivocally define an initial care provider: 1) GPs prior to 2) medical specialists prior to 3) ED outpatient service prior to 4) ED inpatient service.

### Statistical analysis

Statistical analysis was carried out with Stata 15.1 SE and figures were produced using the user-written command *coefplot* [17]. We calculated probabilities for involvement in the care pathway and for the provision of initial care for the different care providers, as well as for the various roles GPs play in the whole care pathway. To consider potential effects of a changing patient population or injury types, we also calculated model-based adjusted probabilities for the outcomes “provider involvement” and “initial care provider” using multinomial logistic regression models controlling simultaneously for patients’ gender, citizenship, age, place of residence, the anatomical location of the injury, and the point in time of the accident. Multinomial logistic regression allows for modelling multiple, unordered nominal outcomes and is a straightforward extension of the logistic regression for binomial outcomes [18]. We report adjusted predicted probabilities which were computed using Stata’s *margins* command [19]. We do not report confidence intervals or *p*-values in the text nor in the figures because, due to our very large sample size, confidence intervals are typically very small. All the reported differences are statistically significant at the 1 % level (two-sided).

## Results

The base data set for our study contains more than 3 million cases registered from 2008 to 2014. The data set used for our main analysis comprises *N* = 2,007,513 accidents that required outpatient medical care but no hospital inpatient stay. The number of included and excluded cases are reported in the supplementing information S 1.

Injuries suffered by patients with an outpatient stay only are mostly of low to moderate severity, as can be indirectly inferred by the typically short incapacity duration and the average low case costs (see descriptives in Table 1). Most of the accidents (58%) resulted in no work absence or in an incapacity duration of three working days or less. 32% of the cases resulted in 4 to 10 working days absence, 8% in 2 weeks to 3 months, and only 2% in more than 3 months. The median of reimbursed direct medical costs per accident was 352 CHF (mean: 806 CHF; 75th percentile: 804 CHF). Hence, the provided medical care consisted in half of the cases of just one or two visits to a healthcare provider performing minor treatments.

**Table 1:** Characteristics of analysis samples. Mean and standard deviation (in parentheses) for numerical variables, proportions for categorical variables

	Main analysis sample outpatient cases only *N* = 2,007,513	Supplemental analysis sample including inpatient cases *N* = 2,195,559
Direct medical costs (CHF, first 24 months)	806 (SD = 1448)	2092 (SD = 10,215)
Incapacity duration		
3 days or less	57.6%	53.2%
More than 3 days	14.5%	13.8%
10 days or more	17.5%	17.5%
More than 1 month	8.5%	10.5%
More than 1 quarter	1.8%	4.1%
More than 1 year	0.1%	0.9%
Year		
2008	12.5%	12.6%
2009	13.5%	13.6%
2010	14.3%	14.3%
2011	14.7%	14.7%
2012	14.8%	14.8%
2013	15.0%	15.0%
2014	15.2%	15.1%
Patient’s place of residence		
Urban	58.1%	57.9%
Intermediate	23.3%	23.3%
Rural	18.6%	18.7%
Patient’s gender		
Male	80.2%	80.3%
Female	19.8%	19.7%
Patient’s citizenship		
Non-Swiss	26.7%	26.4%
Swiss	73.3%	73.6%
Patient’s age	36.8 (SD = 12.6)	37.1 (SD = 12.7)
Occupational vs. non-occupational accident		
Occupational	40.8%	39.7%
Non-Occupational	59.2%	60.3%
Anatomical location of injury		
Cranium	3.3%	3.6%
Eye	9.2%	8.5%
Knee	9.2%	10.4%
Face, nose, ear, jaw	4.0%	3.9%
Shoulder, upper arm	6.6%	7.4%
Lower arm, elbow	3.8%	3.9%
Wrist, hand, finger	25.1%	23.9%
Lower leg, ankle, foot	21.6%	21.2%
Pelvis, hip, thigh, abdomen	4.2%	4.2%
Torso, back, spine, neck	10.8%	10.5%
Other, multiple	2.2%	2.5%
Injury type		
Fracture	6.7%	8.6%
Contusion	23.3%	22.5%
Foreign body	6.8%	6.3%
Sprain, strain	22.3%	22.4%
Bite, burn, abrasion	6.1%	5.8%
Cut	12.3%	11.6%
Other	22.4%	22.8%

80% of the patients in our sample are male, only 20% female – which reflects the fact that the insurance provider from which our data stem has a particularly strong client base in the secondary sector (e.g., manufacturing, construction, infrastructure), where females are employed underproportionally. The mean age is 37 years, about 3 years less than that of the total Swiss workforce; the share of non-Swiss, with 27%, is at the level of the total workforce.

In 4.1% of all cases, more than one group of providers had their first patient contact on the same day, of which 71% were a combination of GP with ED outpatient care, 16% of GP with medical specialist, and 13% of medical specialist and ED outpatient. For these, we assumed a treatment sequence as outlined in the methods section to define an unequivocal initial care provider.

Of those patients receiving care from medical specialists, 35% were treated by orthopedic surgeons, 33% by ophthalmologists, 20% by non-orthopedic surgeons, and 17% by other specialists.

### GPs’ involvement in accident care and initial care provision from 2008 to 2014

**Fig. 1:**
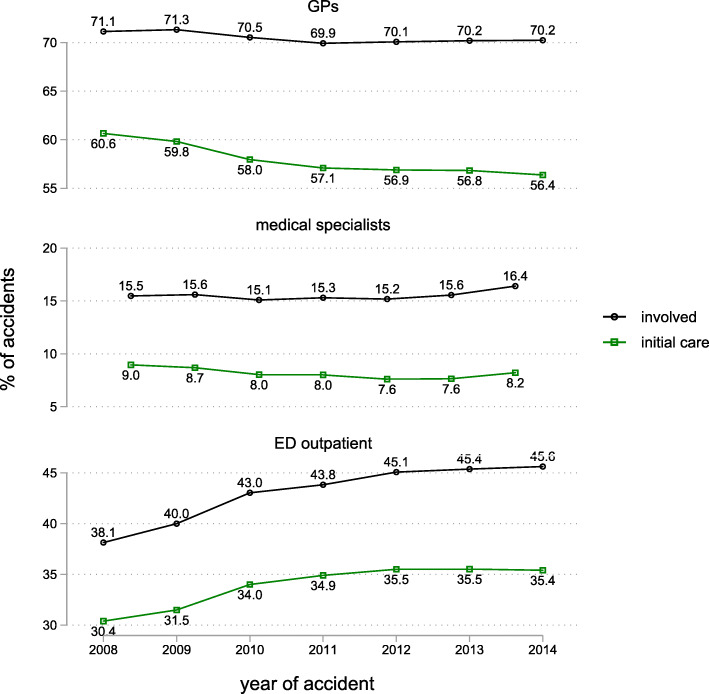
Probability of involvement and of providing initial care for different providers over time. Outpatient cases only, *N* = 2,007,513. Raw probabilities, adjusted probabilities are nearly identical (see Supporting Information S 2). SE of the estimates is always < 0.01 percentage points

In 2014, GPs were involved in 70.2% of all injury cases requiring outpatient medical care but no inpatient stay and provided initial care in 56.4% (Fig. 1). While GPs’ involvement remained almost constant between 2008 and 2014 (drop of − 0.9 percentage points), there was a more pronounced decline by − 4.3 percentage points of GPs providing initial care. For emergency departments (ED), we observe that both involvement (from 38.1 to 45.6%, 7.5 percentage points) and provision of initial care (from 30.4 to 35.4%, 5.0 percentage points) increased substantially. Medical specialists were involved in 16.4% of all cases in 2014 and provided initial care in 8.2% of cases. Over time, involvement increased slightly (+ 0.9 percentage points) while initial care provision declined somewhat (− 0.8%).

### Variations in GPs’ probability of providing initial care by patients’ place of residence, gender, citizenship, age, and point in time of the accident

**Fig. 2:**
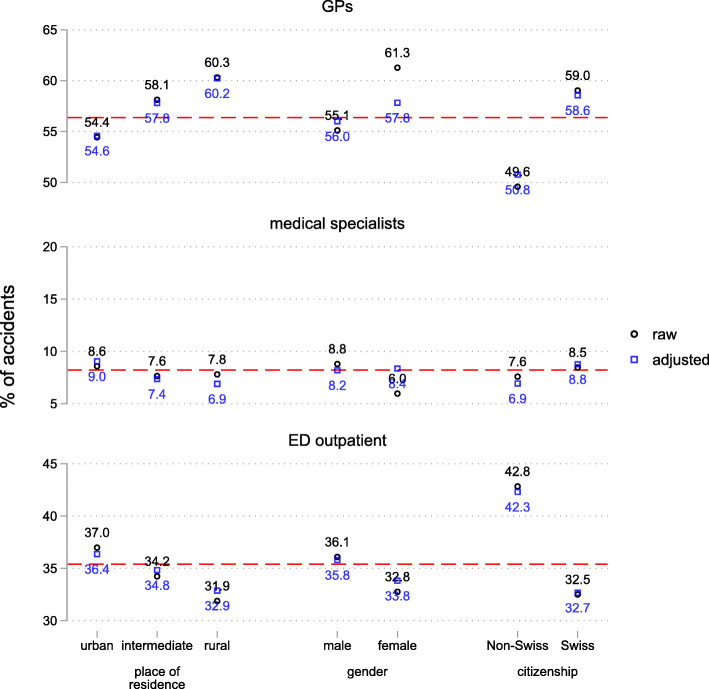
Probability of providing initial care for different providers by patients’ place of residence, gender, and citizenship. 2014, outpatient cases only, *N* = 305,125. Raw and adjusted probabilities, numbers are for raw probabilities. SE of the estimates is always < 0.01 percentage points. The broken red line indicates the mean. Adjusted probabilities are based on a multinomial logistic model controlling for injury type and anatomical location, occupational vs. non-occupational accident, point in time of the accident, patient’s gender, citizenship, age, and place of residence

Whether GPs provide initial care varies substantially between patients residing in urban vs. rural regions (Fig. 2). While in 2014, 54.4% of patients residing in urban regions were provided initial care by GPs, it was 58.1% of patients residing in intermediate, and 60.3% of patients residing in rural regions. EDs show a complementary pattern: the probability that EDs provided initial care was highest in urban regions with 37.0% and lowest in rural regions with 31.9%. Even when adjusting for differences in the patient population, these geographical differences remain.

**Fig. 3:**
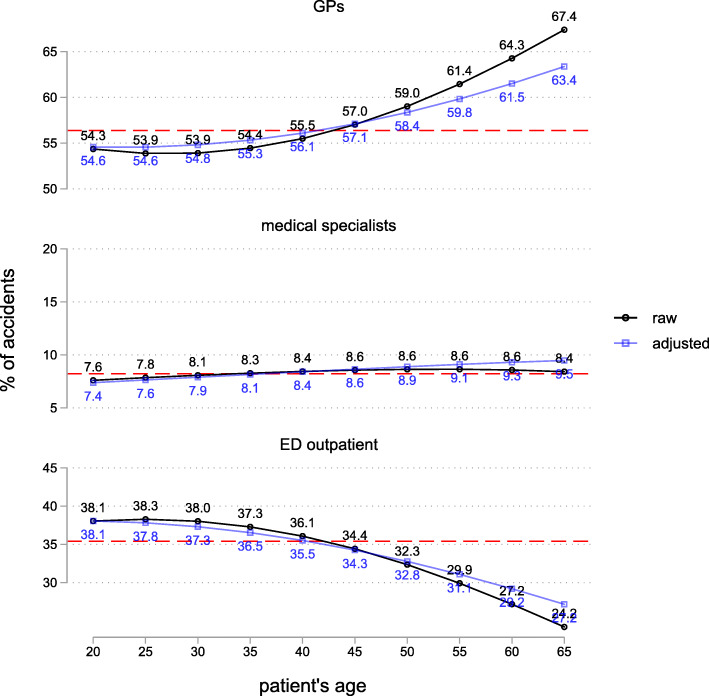
Probability of providing initial care for different providers by patients’ age. 2014, outpatient cases only, *N* = 305,125. Raw and adjusted probabilities. SE of the estimates is always < 0.01 percentage points. The broken red line indicates the mean. Adjusted probabilities are based on a multinomial logistic model controlling for injury type and anatomical location, occupational vs. non-occupational accident, point in time of the accident, patient’s gender, citizenship, age, and place of residence

Males (55.1%) had a somewhat lower probability of seeking initial care from GPs compared to females (61.3%), although the difference diminishes considerably when adjusting for other patient characteristics and injury type (adjusted: 56.1% males vs. 58.0% females). Non-Swiss citizens (49.6%) have a considerably lower probability of seeking initial care from GPs than Swiss citizens (59.0%). The difference decreases somewhat after adjustment but remains large (adjusted: 51.0% Non-Swiss vs. 58.7% Swiss). Finally, younger patients aged 20 to 35 had a lower probability of GPs figuring as initial care provider (about 54%) than elderly patients aged 55+ (above 61%) (Fig. 3). Again, these differences get slightly smaller after covariate adjustment, but remain substantial.

Regarding ED outpatient use, we observe a complementary pattern. Groups with lower probability of receiving initial care from GPs show higher probabilities for ED use.

Concerning medical specialists, there are only minor differences in initial care provision: after adjustment, there is no substantial difference between males and females, a 1.8 percentage points lower probability for Non-Swiss vs. Swiss citizens, and a 2.1 percentage points lower probability for rural vs. urban regions.

**Fig. 4:**
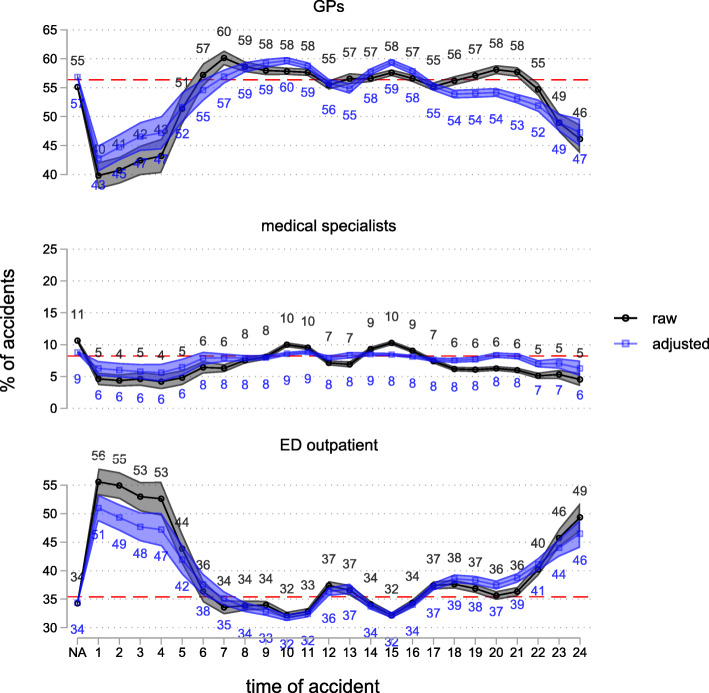
Probability of providing initial care for different providers by time-of-day of the accident. 2014, outpatient cases only, *N* = 305,125. Raw and adjusted probabilities, with 95%-confidence interval. The dashed red line indicates the mean. Adjusted probabilities are based on a multinomial logistic model controlling for injury type and anatomical location, occupational vs. non-occupational accident, point in time of the accident, patient’s gender, citizenship, age, and place of residence.NA: time of accident not available

Further analyses show that there is considerable variation in the provision of initial care depending on the time-of-day and day-of-week the accident occurred. As Fig. 4 shows, accidents occurring during daytime/evening show a considerably higher probability (between 51 and 60%) of GPs providing initial care compared to those happening at night (49% at 11 pm, 40% at 1 am, 43% at 4 am). The probability of GPs providing initial care is also higher for accidents occurring on Sunday (58%) and at the beginning of the week (Monday 59%, Tuesday 58%), compared to those occurring later in the week (Wednesday 56%, Thursday and Friday 55%, Saturday 54%, see figure in Supplementary Information S4). The pattern for EDs is complementary, showing the substitution between GPs and EDs. After adjustment, the differences are less pronounced for time-of-day and slightly more pronounced for day-of-week, showing that the type of accidents and injuries varies substantially according to the point in time the accident occurred. Even after adjustment, considerable injury heterogeneity may remain, which prevents us assigning the observed variations in initial care provision unequivocally to the point in time of the accident.

### Changes in the role of GPs in the care pathway from 2008 to 2014

Who provides initial care after an accident (GPs, medical specialists or a hospital ED) determines strongly the role of GPs during the subsequent care pathway. In the following, we categorize care pathways by GPs’ role in the whole care pathway:

- GP sole care provider: GPs were the only care provider
- GPs → specialists/ED outpatient: initial care by GPs, subsequent care by medical specialists or emergency department (outpatient)
- GP follow-up only: GPs provided only follow-up care after a medical specialist or an ED provided initial care
- GP not involved: GPs were not involved at all

**Fig. 5:**
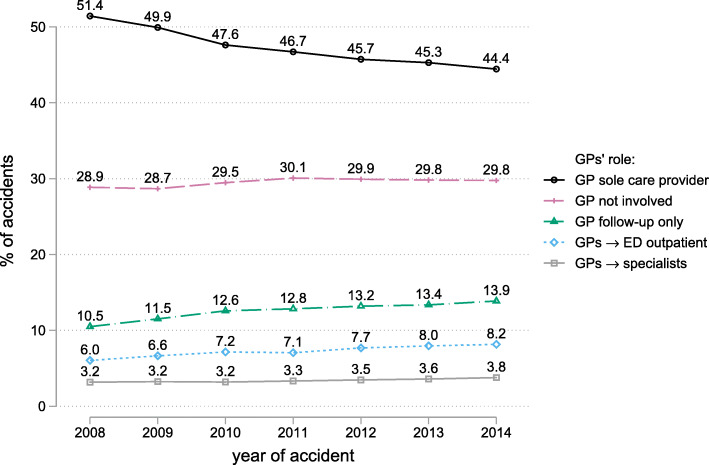
Probability for various roles of GPs in the care pathway over time. Outpatient cases only, *N* = 2,007,513. Raw probabilities. SE of the estimates is always < 0.01 percentage points. GPs → ED outpatient: initial care by GPs and subsequent care by emergency department (outpatient)GPs → specialists: initial care by GPs and subsequent care by medical specialists

In 2014, GPs were the sole care provider in 44.4% of the cases, i.e. no other provider group than GPs was involved (Fig. 5). This is a substantial drop by 7.0 percentage points compared to 2008 when this share was 51.4%. Correspondingly, the share of cases where GPs provided follow-up-only care increased by 3.4 percentage points from 10.5 to 13.9%. Also, the share of cases where GPs provided initial care, whereafter patients saw an ED, increased from 6.0 to 8.2%, while cases where patients subsequently saw a medical specialist remained stable at a low level (2008: 3.2%, 2014: 3.8%). The share of cases GPs are not involved in remained almost unchanged (2008: 28.9%, 2014: 29.8%).

Extending the above analyses of the involvement of the various providers, the initial care provider, and the role of GPs to subgroups of injuries shows that the observed pattern applies to a wide range of injuries (see Supplementing Information S 3). While the percentage of GPs providing initial and sole care is in general higher for less severe types of injuries such as bites or cuts compared to more severe ones such as fractures, the trend of an increasing substitution of EDs for GPs is observable for all injuries considered. The decline in GPs providing initial and/or sole care and the complementary increase in Eds’ involvement is, however, more pronounced for less severe injuries compared to more severe ones (see Supplementing Information S 3).

## Discussion

### Main results

Overall, our results reveal a very important role for GPs in accident care. In 2014, GPs were involved in 70% of all injury cases requiring medical care but no inpatient stay and figured as initial care provider in 56% of them. While involvement stayed at about the same level from 2008 to 2014, cases in which GPs figured as initial care providers have decreased by 4 percentage points during that period. At the same time, accident cases involving care from an emergency department (ED) increased from 38 to 46% and the share receiving *initial* care from an ED from 30 to 35% – apparently substituting for the declining involvement of GPs in initial care. Because of the decline in providing initial care, GPs acted less as sole care providers (decrease from 51 to 44%) and increasingly as follow-up carers only (increase from 10 to 14%). Also, patients who received initial care from GPs increasingly visit EDs or a medical specialist for follow-up care, which indicates that GPs have become more inclined to refer patients to other providers.

While GPs’ general involvement in accident care remained stable over time, they acted less and less as initial care provider. Apparently, EDs stepped in as a replacement resulting in the observed complementary increase in both involvement and initial care provision by EDs. Neither an increase in the non-Swiss population nor an increase in people living in urban areas can explain these changes over time because adjusting for these factors barely affects the trend identified (see Supporting Information S2 for a comparison of raw and adjusted estimates). The share of non-Swiss in the analysis sample only slightly increased from 26 to 28% between 2008 and 2014; the urban-rural ratio stayed constant.

We also found considerable variation in GPs’ role by region and patient characteristics: GPs are less involved in accident care in more urban compared to more rural regions. Males, younger patients, and non-Swiss citizens have a lower probability of receiving initial care from GPs and – as a result – also show a lower probability of having GPs as their sole care provider.

### Comparison with related literature

Our results are consistent with other studies on the topic that report that patients in Switzerland are increasingly treated in hospital EDs [6, 7]. Our study provides systematic evidence of this development from 2008 to 2014 and clearly shows that this is at the cost of GPs, who have been providing less accident care, especially as initial care provider. Hence, Switzerland seems to increasingly suffer from “ED crowding” [20] as most countries in the industrialized world do [21]. Similar developments in the changing role of GPs have been reported for other countries with a comparable health care system relying on a strong GP-led primary care network such as The Netherlands [9] or Germany [22].

Regarding regional variations and differences between patient groups, our results are in line with existing findings from both Switzerland and abroad showing that, for instance, in urban regions, patients rather seek help directly at EDs because EDs are more accessible and convenient [22, 23]. Younger people, as well as non-nationals, might – on average – be less attached to their GPs or might not even have a personal GP, which leads to them seeking help at an ED rather than at GPs’ practices when they need care urgently after an accident [24].

### Implications

Our results provide detailed evidence of a profound and rapid ongoing change regarding GPs’ role in accident care. Potential causes include: changing patient behavior; GPs’ changing skills, preparedness and willingness to treat accident patients; structural factors such as GPs’ opening-hours and availability; and the introduction of new or the more frequent application of special diagnostic tools that are not at easily at GPs’ disposal. In our view, rather than just changing patients’ preferences alone, also structural reasons, such as a change in the way healthcare services are operated and provided to patients, are important drivers of the observed development. The rapid pace of the development suggests this, as does the finding that changes in the patient population over time, such as an increase in urban residents or non-Swiss citizens, are not a driving force behind it.

The fact that the time-of-day and day-of-week of an accident substantially influence whether GPs act as initial care provider indicates that the choice of the initial care provider is shaped by the actual or perceived availability of GPs. One strategy to improve both the actual as well as the perceived out-of-hour availability of GPs is the cooperation of GP-networks with hospitals to create hospital-integrated primary care emergency centers. GP-led, hospital-integrated primary care centers have the potential to improve service quality for patients [25], to increase the job satisfaction of GPs with respect to their out-of-hours duty periods [26, 27], and to reduce costs in hospital EDs [13]. The introduction of primary care physician cooperatives located within hospital EDs led to a sizeable reduction in ED use in the Netherlands [28]. Extending GPs’ opening hours to up to 12 h per day reduced the inappropriate ED use in Italy’s Emilia-Romagna Region by between 10 and 15% [29].

The substantial substitution of services provided by GPs with EDs might impact the quality of care, patient satisfaction and health care costs. A preliminary cost-increase-decomposition (Blinder-Oaxaca, not reported) shows that about a third of the average costs-per-case increase for outpatient cases from 768 CHF (2008) to 853 CHF (2014) is associated with the changing role of GPs.

There are also implications for the education of future GPs. Despite their decreasing involvement in accident care overall, GPs are still confronted with a wide range of injuries and need the corresponding traumatological skills (sutures, treatment of fractures). Our data can contribute to the design of educational tracks that are “evidence-based”, tailored to the most frequently treated injury patterns by GPs [30].

### Strengths and limitations

Our administrative data has some clear advantages over, for instance, self-reported data from population surveys: the data generating process was quite constant over the analysis period and the variables of interest are based on claims and, hence, very valid. Compared to hospital data on ambulatory and inpatient care, our data has the major advantage that it allows for reconstructing care pathways related to one particular accident. Lastly, we have information on all types of providers and can clearly show how, for instance, the decline in GPs’ involvement is related to an increase in care provided by EDs.

Our study has several limitations. First, because of only partially reported data on patients’ beginning of hospital inpatient stays prior to 2014, we restricted the analysis to accidents with no inpatient stay at any point during the care pathway. Accidents with no inpatient stay account for 91.5% of all accidents and its share remained quite stable during the analysis period (91.1% in 2008, 92.0% in 2014, see Figure 8 in the Supporting Information). Also, GPs, the focus of the analyses, provide initial care mostly to outpatients. Results on all patients including inpatient cases, where possible, are reported in the Supporting Information S 5. Our main conclusions regarding the changing GPs’ role are supported in these analyses. Second, we have no information about the reasons why patients are treated by a particular provider. We do not know whether it was the patients’ choice to seek care directly at an ED, whether they were told to do so by their GPs when trying to arrange an appointment, or whether their GPs simply weren’t available. Third, patients’ care pathways could only partially be reconstructed because we only know the date of the initial treatment of a provider group (GPs, medical specialists, EDs). Fourth, our information regarding the patient’s injury is based on self-administered accident report forms and might not always be completely accurate. Consequently, there might be some unobserved injury heterogeneity we cannot control for in our analysis. However, it can reasonably be assumed that, over the analysis period, overall injury patterns did not change substantially. Hence, changes in service provision are likely not due to an (unobservable) change in the composition of the injuries. Fifth, regarding differences in patients’ behavior, we, unfortunately, have no information on relevant patient characteristics such as education, professional background, family situation, or the existence or not of a personal GP. Finally, our results on the SUVA insured are not straightforwardly generalizable to the general population because our data includes only the working population, and no patients younger than 18 and older than 65 years. In addition, patients from the service sector are underrepresented, which leads to an underrepresentation of female employees in our analysis sample. As we have shown, females in our sample have a considerably lower rate of ED use (2014, outpatients only: 33% females vs. 36% males, see Fig. 2). For both males and females, however, there is an identical decline of 5 percentage points between 2008 and 2014, as additional analyses show. Hence, while there is a difference in levels, the observed trend over time is the same for both genders.

## Conclusion

GPs play a key role in accident care with considerable variation depending on region and patient profile. From 2008 to 2014, there is a remarkable decline in GPs’ provision of initial care after an accident at the cost of emergency departments. This is a strong indication that the GPs’ role in the Swiss healthcare system is changing, which may have implications for their continuing education and training as well as for healthcare costs.

## Supplementary information

**Additional file 1.**

## Data Availability

The insurance claims data we use for the analysis was provided by the Swiss National Accident Insurance Fund, Lucerne, Switzerland. It is not publicly available due to privacy concerns and legal restrictions.

## Abbreviations

CHF: Swiss franc
GP: General praticioner
ED: Emergency department
SD: Standard deviation
SE: Standard error
SUVA: Swiss National Accident Insurance Fund

## References

[1] Djalali S, Meier T, Hasler S (2015). Primary care in Switzerland gains strength. Fam Pract.

[2] Künzi K (2005). "Grundversorgungsmedizin" in der Schweiz: Stand der Diskussionen zur Frage der" Grundversorger/innen/Hausärzt/innen" und ihrer zahlenmässigen Entwicklung.

[3] De Pietro C, Camenzind P, Sturny I (2015). Switzerland: health system review. Health Syst Transit.

[4] Tschudi P, Rosemann T (2010). Die Zukunft der Hausarztmedizin! Wie finden wir den Nachwuchs? Womit können wir junge Ärztinnen und Ärzte für das Weiterbildungsziel "Hausärztin" motivieren?. PrimaryCare.

[5] SSUV (2018). Sammelstelle für die Statistik der Unfallversicherung UVG. Unfallstatistik UVG 2017.

[6] Chmiel C, Huber CA, Rosemann T (2011). Walk-ins seeking treatment at an emergency department or general practitioner out-of-hours service: a cross-sectional comparison. BMC Health Serv Res.

[7] Eichler K, Imhof D, Chmiel C (2010). The provision of out-of-hours care and associated costs in an urban area of Switzerland: a cost description study. BMC Fam Pract.

[8] Diserens L, Egli L, Fustinoni S (2015). Emergency department visits for non-life-threatening conditions: evolution over 13 years in a Swiss urban teaching hospital. Swiss Med Wkly.

[9] Minderhout RN, Venema P, Vos HMM (2019). Understanding people who self-referred in an emergency department with primary care problems during office hours: a qualitative interview study at a daytime general practice cooperative in two hospitals in the Hague, The Netherlands. BMJ Open.

[10] Finkenstädt V. Die ambulante ärztliche Versorgung in Deutschland, den Niederlanden und der Schweiz. WIP-Diskusisonspapier. 2015;4.

[11] Merçay C (2015). Médecins de premier recours – Situation en Suisse, tendances récentes et comparaison internationale.

[12] Cohidon C, Cornuz J, Senn N (2015). Primary care in Switzerland: evolution of physicians' profile and activities in twenty years (1993-2012). BMC Fam Pract.

[13] Eichler K, Hess S, Chmiel C (2014). Sustained health-economic effects after reorganisation of a Swiss hospital emergency Centre: a cost comparison study. Emerg Med J.

[14] Hugentobler W (2006). Kostenvergleich der ambulanten Notfallversorgung in der hausärztlichen Praxis mit den Notfallstationen der Spitäler. PrimaryCare.

[15] Fritschi CB, Ballmer PE (2014). Vergleich der Betreuung ambulanter Notfall-patienten in der hausärztlichen Praxis und dem Zentrumsspital. Praxis.

[16] BFS Bundesamt für Statistik (2017). Raumgliederungen der Schweiz. Gemeindetypologie und Stadt/Land-Typologie 2012. BFS Aktuell.

[17] Jann B (2014). Plotting regression coefficients and other estimates. Stata J.

[18] Long JS (1997). Regression models for categorical and limited dependent variables.

[19] Williams R (2012). Using the margins command to estimate and interpret adjusted predictions and marginal effects. Stata J.

[20] Moskop JC, Sklar DP, Geiderman JM (2009). Emergency department crowding, part 1 - concept, causes, and moral consequences. Ann Emerg Med.

[21] Berchet C (2015). Emergency Care Services.

[22] Schmiedhofer M, Möckel M, Slagman A (2016). Patient motives behind low-acuity visits to the emergency department in Germany: a qualitative study comparing urban and rural sites. BMJ Open.

[23] Krämer J, Schreyögg J (2019). Substituting emergency services: primary care vs. hospital care. Health Policy.

[24] Ruud SE, Hjortdahl P, Natvig B (2017). Reasons for attending a general emergency outpatient clinic versus a regular general practitioner – a survey among immigrant and native walk-in patients in Oslo, Norway. Scand J Prim Health Care.

[25] Wang M, Wild S, Hilfiker G (2014). Hospital-integrated general practice: a promising way to manage walk-in patients in emergency departments. J Eval Clin Pract.

[26] Huber CA, Rosemann T, Zoller M (2011). Out-of-hours demand in primary care: frequency, mode of contact and reasons for encounter in Switzerland. J Eval Clin Pract.

[27] Hess S, Sidler P, Chmiel C (2015). Satisfaction of health professionals after implementation of a primary care hospital emergency Centre in Switzerland: a prospective before–after study. Int Emerg Nurs.

[28] Smits M, Rutten M, Keizer E (2017). The development and performance of after-hours primary Care in the Netherlands: a narrative review. Ann Intern Med.

[29] Lippi Bruni M, Mammi I, Ugolini C (2016). Does the extension of primary care practice opening hours reduce the use of emergency services?. J Health Econ.

[30] Höglinger M, Knöfler F, Schaumann-von Stosch R, et al. Unfallversorgung durch die Hausarztpraxis: zentrale Anlaufstelle, breites Verletzungsspektrum. Suva Medical. in press.

