# Supplementary material for: Recent trends and variations in general practitioners’ involvement in accident care in Switzerland: an analysis of claims data
Source: BMC Fam Pract. 2020 Jun 5;21:99. doi: 10.1186/s12875-020-01170-5 (PMC7275559; doi:10.1186/s12875-020-01170-5)
Supplement: Supplementary file 1 — Additional file 1. [file 12875_2020_1170_MOESM1_ESM.docx]

Supporting Information

1. Tables describing the analysis samples

Table 1: Number of cases included in the study

|  |  |  |
| --- | --- | --- |
| All claims registered 2008-2014 | | 3,096,618 |
| Exclusion | patient residence abroad | 194,048 |
|  | patient age 65 or more | 19,994 |
|  | patient age below 18 | 141,860 |
|  | dental lesion as main injury | 56,191 |
|  | date of accident prior to 2008 | 32,776 |
|  | claim registration later than 90 days after accident | 177,230 |
|  | treatment abroad | 2,226 |
|  | no treatment bills | 66,197 |
|  | none of the considered provider groups involved (GPs, medical specialists, ED in- and outpatient) | 210,537 |
| Total including cases with inpatient stay (supplemental analysis sample) | | 2,195,559 |
| Total excluding cases with inpatient stay (main analysis sample) | | 2,007,513 |

1. Results over time with covariate adjustments

Figure 1: Probability of involvement for different care providers over time. Outpatients only, N=2,007,513. Raw and adjusted probabilities. SE of the estimates is always <0.01 percentage points. Adjusted probabilities are based on a multinomial logistic model controlling for injury type and anatomical location, occupational vs. non-occupational accident, point in time of the accident, patient’s gender, citizenship, age, and place of residence.

Figure 2: Probability of providing initial care for different providers over time. Outpatient cases only, N=2,007,513. Raw and adjusted probabilities. SE of the estimates is always <0.01 percentage points. Adjusted probabilities are based on a multinomial logistic model controlling for injury type and anatomical location, occupational vs. non-occupational accident, point in time of the accident, patient’s gender, citizenship, age, and place of residence.

1. Provider involvement, initial care provision, and GPs’ role by selected injuries

Figure 3: Probability of involvement for different care providers over time by selected injuries. Outpatient cases only. Raw probabilities. fx: fracture

Figure 4: Probability of providing initial care for different providers over time by selected injuries. Outpatient cases only. Raw probabilities. fx: fracture

Figure 5: Probability of various roles of GPs in care pathway over time by selected injuries. Outpatient cases only. Raw probabilities.
GPs 🡺 ED outpatient: initial care by GPs and subsequent care by emergency department (outpatient)
GPs 🡺 specialists: initial care by GPs and subsequent care by medical specialists

1. Initial care provider by day-of-week of the accident

Figure 6: Probability of providing initial care for different providers by day-of-week of the accident. 2014, outpatient cases only, N=305,125. Raw and adjusted probabilities. SE of the estimates is always <0.01 percentage points. The dashed red line indicates the mean. Adjusted probabilities are based on a multinomial logistic model controlling for injury type and anatomical location, occupational vs. non-occupational accident, point in time of the accident, patient’s gender, citizenship, age, and place of residence.

1. Results including inpatient cases

Because of only partially reported data on patients’ begin of inpatient stays prior to 2014, we have to restrict analyses on initial care providers and on GPs’ role to the year 2014 if we include inpatient cases. Only for provider involvement, we can analyze the whole period from 2008 to 2014 (Figure 8).

Figure 7: Probability of involvement and of initial care provision for different care providers, and of the various roles of GPs in the care pathway. All patients, 2014, N=331,786. SE of the estimates is always <0.01 percentage points.

Figure 8: Probability of involvement for different care providers over time. All patients, N=2,195,013. SE of the estimates is always <0.01 percentage points.

Figure 9: Probability of providing initial care for different providers by patients’ place of residence, gender, and citizenship. All patients, 2014, N=331,786. Raw and adjusted probabilities. SE of the estimates is always <0.01 percentage points. The dashed red line indicates the mean. Adjusted probabilities are based on a multinomial logistic model controlling for injury type and anatomical location, occupational vs. non-occupational accident, point in time of the accident, patient’s gender, citizenship, age, and place of residence.
